# Bibliography

**Published:** 1896-01

**Authors:** 


### Bibliography.

Kronen und Brücken-Arbeiten. Ein Lehrbuch von Hans Riegner, Dr. Chir. Dent., Zahnarzt in Breslau. Erste Halfte, mit 266 Illustrationen. Verlag von Arthur Felix. Leipzig, 1895.

It is impossible to open the first half of this finely-illustrated and well-prepared work of Dr. Riegner on "Crowns" without a feeling that, whatever may be said of dentistry, as a whole, in its relation to American inventions, the proof is here that in crown-work, at least, other countries make but a poor showing in comparison.

The author has, with marked ability and industry, gathered all that has been written upon this subject, and that with the thoroughness peculiar to the German character, yet in this first part he has not been able to get beyond the American continent to any extent.

After giving a very clear description of the treatment and preparation of teeth and roots, the author begins the consideration of porcelain crowns. He regards Bonwill as one of the earliest advocates of the porcelain-crown system, and he therefore gives his crown the place of honor, the first to be described. While all credit is due to Dr. Bonwill, it should not be forgotten that crowns were made more than a half-century ago; but, while this is true, it does not materially alter the fact that the all porcelain crown of Bonwill had and still continues to have a marked place in dentistry.

From Bonwill he passes on to Howe, Gates, Foster, Genese, Stowell, Howland, Logan, Richmond, Brown, Weston, Meriam, Perry, Kirk, Townsend, Büttner, Richardson, Parr, Leech, Low, and Sachs. This is a fairly representative list, and exhibits an industrious search through the literature of this subject. It is questionable whether some have sufficient originality to warrant embalming in a work of this character.

The preparation of the roots for crowns is clearly demonstrated both in the text and by illustrations. The third part, on "Bridge-Work," will appear as a second volume. This seems to be a mistake, as these matters are so intimately related they should go together. It is possible that, as this is issued in paper cover, it may be supposed readers will have them bound together. This is not, however, the American habit.

The illustrations and general make up are worthy the house of Arthur Felix, the publications of which have a deserved reputation everywhere.

ANNUAL OF THE UNIVERSAL MEDICAL SCIENCES. A Yearly Report of the Progress of the General Sanitary Sciences throughout the World. Edited by Charles E. Sajous, M.D., and Seventy Associate Editors, assisted by over two hundred Corresponding Editors, Collaborators, and Correspondents. Illustrated with Chromo-Lithographs, Engravings, and Maps. The F. A. Davis Company, Publishers, Philadelphia, New York, Chicago, London, 1895.

The editor of the Annual in his preface apologizes for the late appearance of these five volumes, in that "his best efforts to insure the 1895 series an early appearance were again frustrated by the tardy arrival of several papers." It is presumed that those who look forward with interest to this annual report will willingly excuse the editor, for there are few, it is imagined, but would prefer to wait to have the work presented in the satisfactory manner with which this collection is given

It is not surprising that this annual report should have had a remarkable success; indeed, it seems to the writer no one can claim to be fully abreast with the world's work in medicine without it, for it is not to be presumed that any one individual could possess the facilities for condensing this possessed by Dr. Sajous and his assistants.

As a work of reference in all branches of medicine it has no equal; in fact, it is believed no similar publication exists in which an attempt is made, and that successfully, to compass all branches of the healing art, excepting dentistry, in an annual series of volumes. This latter defect is neither the fault of the editor nor of the publishers, for every effort was made to have this subject included, but, unfortunately for this work and dental literature, the practice of this specialty is so exhausting as to preclude the possibility of an extended range of authorship. Since this publication was begun there has been a marked change in the division of work, with a decided increase in the number of those willing and able to write. It would, therefore, seem to be a favorable period for beginning a chapter or chapters on dental work.

The nearest approach to this specialty is the chapter on "Oral Surgery," ably conducted by Rudolph Mates, M.D., of New Orleans.

In the chapter on Anæsthetics, by Dudley Buxton, M.D., B.S. (Lond.), he devotes considerable space to "the discovery of anæsthesia," in which he quotes Dr. McManus and others, and summarizes the history fairly, but with special skill in avoiding committing himself to one side or the other in the prolonged controversy. He, however, concedes that Morton was the one to make the "first systematic attempt to employ ether in general surgery." This article, as a whole, is of special interest.

It would be a pleasure to follow the work of other editors and collaborators, but space will not permit.

No medical library, personal or public, can be complete without this series of volumes, and it is a pleasure to feel that both editors and publisher are receiving satisfactory returns in every way for the great labor bestowed in their preparation.

